# Protein binding hot spots and the residue-residue pairing preference: a water exclusion perspective

**DOI:** 10.1186/1471-2105-11-244

**Published:** 2010-05-12

**Authors:** Qian Liu, Jinyan Li

**Affiliations:** 1 Bioinformatics Research Center & School of Computer Engineering, Nanyang Technological University, Singapore 639798

## Abstract

**Background:**

A protein binding hot spot is a small cluster of residues tightly packed at the center of the interface between two interacting proteins. Though a hot spot constitutes a small fraction of the interface, it is vital to the stability of protein complexes. Recently, there are a series of hypotheses proposed to characterize binding hot spots, including the pioneering O-ring theory, the insightful 'coupling' and 'hot region' principle, and our 'double water exclusion' (DWE) hypothesis. As the perspective changes from the O-ring theory to the DWE hypothesis, we examine the physicochemical properties of the binding hot spots under the new hypothesis and compare with those under the O-ring theory.

**Results:**

The requirements for a cluster of residues to form a hot spot under the DWE hypothesis can be mathematically satisfied by a biclique subgraph if a vertex is used to represent a residue, an edge to indicate a close distance between two residues, and a bipartite graph to represent a pair of interacting proteins. We term these hot spots as DWE bicliques. We identified DWE bicliques from crystal packing contacts, obligate and non-obligate interactions. Our comparative study revealed that there are abundant *unique *bicliques to the biological interactions, indicating specific biological binding behaviors in contrast to crystal packing. The two sub-types of biological interactions also have their own signature bicliques. In our analysis on residue compositions and residue pairing preferences in DWE bicliques, the focus was on interaction-preferred residues (ipRs) and interaction-preferred residue pairs (ipRPs). It is observed that hydrophobic residues are heavily involved in the ipRs and ipRPs of the obligate interactions; and that aromatic residues are in favor in the ipRs and ipRPs of the biological interactions, especially in those of the non-obligate interactions. In contrast, the ipRs and ipRPs in crystal packing are dominated by hydrophilic residues, and most of the anti-ipRs of crystal packing are the ipRs of the obligate or non-obligate interactions.

**Conclusions:**

These ipRs and ipRPs in our DWE bicliques describe a diverse binding features among the three types of interactions. They also highlight the specific binding behaviors of the biological interactions, sharply differing from the artifact interfaces in the crystal packing. It can be noted that DWE bicliques, especially the unique bicliques, can capture deep insights into the binding characteristics of protein interfaces.

## Background

A protein binding hot spot is a small cluster of residues [1] tightly packed at the center of the interface between two interacting proteins. Though a hot spot constitutes a small fraction of the interface, it contributes most to the binding stability and free energy. A hot spot of binding energy was initially conceptualized by Clackson and Wells (1995), supported by an important finding that a 'functional epitope' (a hot spot) between human growth hormone and its receptor accounts for more than three-quarters of the binding free energy [2]. This hot spot was also found to be geometrically surrounded by less important contact residues that are generally hydrophilic and partially hydrated [2]. On the basis of these pioneering observations and studies, Bogan and Thorn (1998) formalized more intuitively a hypothesis named O-ring theory to characterize the topological shape of the surrounding residues [1]. The O-ring theory points out that the residues of the O-ring likely function a role to occlude bulk water molecules from the hot spots. Thus, the O-ring theory is also known as 'water exclusion' hypothesis [1,3-8]. This theory is profound and influential. However, the organizational topology of the ring-inside, energetically more important hot spot residues is not specified by the O-ring theory. Recently, we investigated the spatial adjacency and vicinity of these hot spot residues and proposed a hypothesis called 'double water exclusion' (DWE) [9] to refine the O-ring theory. At one hand, the DWE hypothesis agrees with the O-ring principle that there should exist a ring of residues surrounding the hot spot for avoiding the invasion of water molecules after the complex formation; on the other hand, the DWE hypothesis affirms that the hot spot itself is water-free, having a zero-tolerance to water molecules. In fact, the DWE hypothesis shares a light with the 'coupling' proposition [10] which is another insightful theory about hot spot residues, and it also theoretically strengthens the influential 'hot region' principle [11]. The requirements for a cluster of residues to form a hot spot under the DWE hypothesis can be mathematically satisfied by a biclique subgraph [9] if a vertex is used to represent a residue, an edge to indicate a close distance between two residues, and a bipartite graph to represent a pair of interacting proteins. We term these hot spots as DWE bicliques, and note that in a DWE biclique, residues from one chain all have full connection with the residues from the other chain. In our latest evaluation [9] by applying to the ASEdb repository [4] and the Hotsprint database [12], we found that these DWE bicliques are rich of true hot spot residues.

With the perspective change from the O-ring theory to the DWE hypothesis, it is interesting to study the physicochemical properties of DWE biclique hot spots, and to compare with those [1,3,13] under the O-ring theory. Specifically in this paper, we examine those DWE bicliques that are unique to crystal packing contacts, or unique to biological interactions including obligate and non-obligate interactions. Crystal packing are enforced by crystallographic packing environments and formed during the crystallization process, but they do not occur in solution or in their physiological states [14]. On contrast, obligate interactions are stable, but their protein chains have no stable tertiary structures *in vivo *and they function only in the complex form [15]. However, the protomers in non-obligate interactions may disassociate after the accomplishment of a particular function [15]. Clearly, obligate and non-obligate interactions depend on various factors for promoting complex formation although some factors are common, while the non-specific crystal packing should have different properties from these two kinds of biological interactions. So at the residue level, it is of our primary interests to see which DWE bicliques are signature binding hot spots of the different types of protein interactions, and which are common for demonstrating their such difference. Given two types of interactions (e.g., biological interactions and crystal packing contacts), a unique DWE biclique is defined as a DWE biclique that frequently occurs in one type of interaction (e.g., biological interactions) but is absent in the other type (e.g., crystal packing). We also examine the residue composition of DWE bicliques, and the distribution of residue pairs and their pairing preference within DWE bicliques.

Residue composition and residue pairing preference are two fundamental physicochemical properties for protein folding [16,17] and protein interactions [18,19]. Both of them have been broadly used in comparative analyses based on protein structure data. One aspect of this comparative analyses is on different segments within protein complex structures, such as comparison between intraproteins and protein interfaces [19], or interdomain comparison [20]; another angle is on different types of protein interactions [13,21,22]. De *et al*. [23] and Lukman *et al*. [24] analyzed residue contacts of obligate complexes and non-obligatory/transient protein complexes, and reported that the interaction patterns of these two types of complexes were different. Ofran and Rost [20] studied the preferences of residue contacts in a more complicate way for six types of protein interfaces. One finding is that the preferences differed remarkably between the six types of interfaces. However, in almost all previous works, the individual residues and the residue pairs were dissected based on the whole interfaces. In this work, we analyze the composition of residues and residue pairs in DWE bicliques, the energetically important part of each interface.

We propose to use interaction-preferred residues (ipRs) and interaction-preferred residue pairs (ipRPs) to describe the binding specificity for different types of interactions. Our important findings include: (i) in the obligate interactions, hydrophobic ipRs and ipRP contacts involving only hydrophobic residues are widely conserved, no anti-ipRs are hydrophobic, and the contacts involving only hydrophilic residues are depleted in ipRPs; (ii) aromatic ipRs and their ipRPs much prefer to the biological interactions, especially to the non-obligate interactions; (iii) hydrophilic ipRs and ipRPs involving only hydrophilic residues seem to be rich in crystal packing contacts; the anti-ipRs of crystal packing, such as Met, Trp and Cys, are just the ipRs of the obligate or non-obligate interactions. So, these ipRs and ipRPs in DWE bicliques provide a clear distinction for the specific binding behaviors of biological interactions as well as crystal packing contacts.

## Results and Discussion

The data used for our evaluation is a nonredundant data set consisting of 291 crystal packing contacts, 289 non-obligate interactions and 287 obligate interactions. This data set is combined from 4 previously compiled datasets [22,25-27] after a redundancy-removal process shown in **Methods**.

We identified 1580, 725 and 208 DWE bicliques from these obligate, non-obligate and crystal packing contacts, respectively. All of these DWE bicliques have at least two residues in the smaller side and at least three residues in the bigger side, and occur in at least two interactions. The detailed computational steps are presented in the **Methods **section for how to detect DWE bicliques from a pair of interaction chains. There are 26 obligate interactions, 75 non-obligate interactions and 159 crystal packing that do not contain any DWE bicliques. Please note that by 'containing no DWE bicliques', we mean that these interactions do not contain any DWE bicliques of high frequency or of big size; they may contain some bicliques with low support (only one occurrence) or with a small size (for example, those bicliques with only one residue from each protein chain).

The remaining results are organized into four parts in this section. Firstly, we show that a binding hot spot is usually a very small area in comparison to its binding interface. Then, we present the distribution of DWE bicliques among the three types of protein interactions to provide an intuitive way for understanding the protein interfaces. After that, we conduct statistical analysis on the composition of the residues and on the pairing preference of residue pairs in our DWE bicliques.

### A. Binding Hot Spots are Small in Binding Interfaces

We calculated the fractions of residues in DWE bicliques over interface residues. As expected, we found that binding hot spots are small in binding interfaces as shown in Table 1. In this table, all the DWE bicliques are from the biological interactions; in the first column, the numbers split by '-' mean that the residue number of the smaller partite in DWE bicliques must be not less than the left number, and that the residue number of the larger partite must be not less than the right number; in the following two columns, *C *means that the fraction is over the whole contact residues, and *I *means that the fraction is over the whole interface residues. Here, interface residues comprise *contact residues *and *their nearby residues *[28]. A pair of contact residues are two residues each from one chain that have at least two atoms contacting to each other. (Two atoms are considered to contact to each other if their distance is below the sum of their van der Waals radii plus 2.75 Å.) The nearby residues refer to those residues that are from the same chain as the contact residues, and their CA atoms also contact with the CA atoms of contact residues. Here, two CA atoms are considered to contact if their distance is less than 6 Å [28].

**Table 1:** The fraction of DWE bicliques over the interfaces.

Minimum Biclique Size	Fraction_*C *_(%)	Fraction_*I *_(%)
1-2	71.99	39.33

2-2	50.23	27.44

2-3	44.69	24.42

### B. Common and Unique DWE Bicliques in Different Types of Interactions

In this work, unique DWE bicliques are defined based on bicliques' *support *information. Given a set of interactions containing one or more types of interactions, a DWE biclique may have multiple occurrence in these interactions. We call the number of occurrence the *support *level of this biclique. We also use *support ratio r *to help the comparisons of DWE bicliques in different types of interactions. Suppose that a biclique has a higher support *S*_*h *_in one type *c*_*h *_and a lower support *S*_*l *_in the contrasting type *c*_*l*_, *r *= *S*_*h*_/*S*_*l *_indicates the preference of this biclique between these two types. In the extreme cases when the support ratio *r *is infinite (i.e., *S*_*l *_= 0), this biclique is more interesting and it is defined as a unique biclique. In other words, this kind of unique bicliques have zero occurrence in one entire type of protein interactions but have multiple, perhaps very high, occurrence in the other type. To avoid some possible noise patterns, we require that *S*_*h *_+ *S*_*l *_should be not less than 2 which is consistent with the biclique filtering constraint presented in **Methods**. Thus for unique bicliques, *S*_*h *_is always equal to or greater than 2 since *S*_*l *_= 0. Obligate and non-obligate interactions are two types of distinct biological interactions, each possessing their specific binding behaviors. However, crystal packing contacts are randomly formed by chance. We present DWE bicliques that are unique to biological interactions (*c*_*p*+_) when compared to crystal packing contacts (*c*_*n*-_), and those unique to crystal packing when compared to biological interactions. Meanwhile, the common bicliques, those with support ratio around 1.0, between biological interactions and crystal packing are also presented. Then we report common and unique DWE bicliques between obligate (*c*_*p*+_) and non-obligate interactions (*c*_*n*-_) to demonstrate their distinct binding specificity. After that, a case study is followed to examine deep structural details of unique DWE bicliques. Here, the symbol *c*_*p*+ _stands for a 'positive' set, while the symbol *c*_*n*- _stands for a 'negative' set. If the higher support *S*_*h *_of a biclique is in the positive set, then we denote the support ratio *r *as *S*_*h*_/*S*_*l*_; otherwise if the higher support *S*_*h *_is in the negative set, we denote *r *as -*S*_*h*_/*S*_*l*_.

#### Common and Unique DWE Bicliques: Biological Interactions vs Crystal Packing Contacts

The distribution of DWE bicliques on the range of their support ratios from -INF to +INF is shown in Table 2. It can be noted that the type of crystal packing contacts contains much fewer favorite DWE bicliques than the type of biological interactions (columns 2 and 3 vs columns 5-8). In particular, there are only 13 DWE bicliques unique to crystal packing, counting to only 0.79% of the total DWE bicliques occurring in the biological and crystal packing interactions. In contrast, the type of biological interactions is rich of DWE bicliques, including unique bicliques. Table 2 shows that about 86.2% bicliques are unique bicliques to biological interactions. Few DWE bicliques contained by crystal packing but abundant DWE bicliques in biological interactions are interpretable with the following reasons: (i) crystal packing are constructed randomly based on protein surfaces during the crystallization process, and their artifact interfaces cannot form much repeatable DWE bicliques; (ii) residues in biological interfaces are found to be more conserved than crystal packing and than the rest of protein surfaces [26,29], and thus biological interactions can easily form stable and repeatable biclique structures.

**Table 2:** Common and unique DWE bicliques in biological interactions and crystal packing.

	(-)crystal packing		biological interactions(+)				
***support *level**	**range of support ratios**						
	
	**-INF**	**-2**	**1**	**2**	**3**	**> = 4**	**INF**
	
	**-INF**	**(-2.5,-1.5]**	**(-1.5,1.5)**	**[1.5,2.5)**	**[2.5,3.5)**	**[3.5,7.5)**	**INF**

2	13	0	152	0	0	0	1144

3	0	3	0	38	0	0	207

4	0	0	0	0	13	0	40

> = 5	0	0	0	1	7	6	23

Total	13	3	152	39	20	6	1414

#### Common and Unique DWE Bicliques: Obligate vs Non-obligate Interactions

Table 3 shows the number of common and unique bicliques for the obligate and non-obligate interactions. About 43.5% (641) of them are unique bicliques for the type of obligate interactions, and 16.6% (244) of them are for the type of non-obligate interactions. These unique bicliques can demonstrate the intuitive difference of binding behaviors in the two types of biological interactions, although there exist overlapping bicliques occurring in both obligate and non-obligate interactions. Detailed information of important unique DWE bicliques will be provided in the next subsection.

**Table 3:** Common and unique DWE bicliques in obligate and non-obligate interactions.

	(-)non-obligate interactions			obligate interactions(+)			
**support level**	**range of support ratios**						
	
	**-INF**	**-3**	**-2**	**1**	**2**	**> = 3**	**INF**
	
	**-INF**	**(-3.5,-2.5]**	**(-2.5,-1.5]**	**(-1.5,1.5)**	**[1.5,2.5)**	**[2.5,5.5)**	**INF**

2	223	0	0	436	0	0	523

3	17	0	38	0	76	0	90

4	2	5	0	6	0	12	19

> = 5	2	0	1	0	3	11	9

Total	244	5	39	442	79	23	641

#### Related Evidence for Unique Bicliques

The existence of unique bicliques in the three types of interactions is in agreement with the observations in our another work [30] for classifying three types of interactions (i.e. crystal packing, obligate and non-obligate interactions). In that work, we compared classification performance between our propensity vectors, binary vectors and frequency vectors on three literature datasets under three evaluation frameworks. In [30], all of the propensity vectors, binary vectors and frequency vectors contain 3-dimensional summary information of protein interactions and another 210 dimensions for residue pairs. The difference was that the values of 210-dimensional residue pairs in binary vectors indicated whether a certain residue pair occurs or not in a certain corresponding protein interaction, and those in frequency vectors were the frequency of corresponding residue pairs in protein interactions, while those in propensity vectors were the propensity value of corresponding residue pairs in protein interactions.

Our comparison results of these three vectors in [30] suggested following two evidences to support our concept of unique bicliques. (i) In almost all cases, the performances under the DWE hypothesis are better than those on all interface residues. That is, the whole interfaces may cover more noise features among different types of interactions, while DWE bicliques can remove some noise patterns and pinpoint distinct features. (ii) Binary vectors had a similar high classification performance to, sometimes a bit higher than, the frequency vectors. That similar performance maybe implied that certain combination patterns of residue pairs rather than their frequency are signature features for different types of protein interactions. When DWE bicliques are detected from DWE bipartites of protein interactions, these DWE bicliques are likely to be this kind of the combination patterns. In fact, the union of bicliques, possibly not maximal bicliques, might also be one kind of the combination patterns.

#### Unique Bicliques: A Case Study

We present four unique DWE bicliques and study their structural properties. They are: (i) a unique biclique  = ⟨{GLY, GLY, LYS}, {GLN, THR}⟩, which occurs only in two crystal packing contacts (Table 4); (ii) a unique biclique  = ⟨{LEU, THR, VAL}, {LEU, VAL}⟩, which occurs only in five obligate interactions as shown in Table 5; a DWE biclique  = ⟨{ALA, LEU, VAL}, {LEU, TYR}⟩ which is a unique biclique to the type of biological interactions occurring in two obligate and three non-obligate interactions (Table 6), and (iv) a unique biclique  = ⟨{GLN, GLY, SER, SER, TYP}, {CYS, LYS}⟩ contained in only 6 non-obligate interactions (Table 7). At the first column of these four tables, the first four letters represent PDB entry identifiers, if necessary, followed by '-' and two interaction protein chains which are separated by ':'. At the columns 2 and 3 of these four tables, the strings split by '-' are residue types followed by their corresponding positions in the amino acid sequences, representing specific residues from the two interacting protein chains.

**Table 4:** Interactions involving the unique biclique  = ⟨{GLY, GLY, LYS}, {GLN, THR}⟩ of crystal packing

PDB entry	First Chains	Second Chains
1BG0	GLY117-LYS235-GLY236	GLN196-THR197

2ACY	GLY15-LYS16-GLY45	GLN44-THR46

**Table 5:** Interactions involving the unique biclique  = ⟨{LEU, THR, VAL},{LEU, VAL}⟩ of obligate interactions.

PDB entry	First Chains	Second Chains
1AD3-A:B	VAL396-THR398-LEU399	VAL86-LEU97

1B7B-A:C	THR109-VAL110-LEU111	VAL110-LEU111

1B8J-A:B	LEU33-THR81-VAL430	LEU80-VAL430

1LDJ-A:B	VAL539-LEU540-THR580	VAL30-LEU32

1QOE-A:B	LEU250-VAL261-THR262	LEU250-VAL261

**Table 6:** Interactions involving the DWE biclique  = ⟨{ALA, LEU, VAL},{LEU, TYR}⟩ occurring in both obligate and non-obligate interactions

PDB entry	First Chains	Second Chains	Interaction Type
1AT3-A:B	LEU214-ALA217-VAL218	TYR124-LEU213	Obligate

1GO3-E:F	VAL12-ALA17-LEU41	TYR2-LEU75	Obligate

1DOA-A:B	VAL36-ALA59-LEU67	LEU48-TYR51	Non-obligate

1EVT-A:C	LEU165-ALA167-VAL168	TYR15-LEU135	Non-obligate

1JSU-A:C	VAL18-ALA31-LEU83	LEU84-TYR88	Non-obligate

**Table 7:** Interactions involving the uniquebiclique  = ⟨{GLN, GLY, SER, SER, TYP},{CYS, LYS}⟩ of non-obligate interactions.

PDB entry	First Chains	Second Chains
1EJA-A:B	GLN192-SER195-SER214-TRP215-GLY216	CYS33-LYS34

1TAB-E:I	GLN192-SER214-TRP215-GLY216-SER217	CYS24-LYS26

1TGS-Z:I	GLN192-SER214-TRP215-GLY216-SER217	CYS16-LYS18

2BTC-E:I	GLN192-SER214-TRP215-GLY216-SER217	CYS503-LYS505

2PTC-E:I	GLN192-SER195-SER214-TRP215-GLY216	CYS14-LYS15

1BTH-H:P	GLN192-SER195-SER214-TRP215-GLY216	CYS14-LYS15

We take these examples to highlight that the uniqueness of DWE bicliques matches to different interfacial properties of the three types of protein interactions in terms of polarity, hydrophobicity, the composition of residues and residue pairs in protein interfaces. As mentioned, the unique biclique  (Table 4) has a support of only 2. In fact, the maximum support of the unique bicliques that occur in the type of crystal packing contacts is 2. While the maximum support of the unique bicliques in the biological interactions is larger, 7 for the obligate bicliques and 6 for the non-obligate bicliques. Thus, the biclique structures in the biological interactions are more stable and repeatable than those in the crystal packing contacts. The four examples of unique bicliques also give a glance at residue composition in the three types of interactions. As shown in Table 4, the unique biclique  of crystal packing consists of more polar and hydrophilic residues, such as GLY/LYS and GLN/THR, while the unique biclique  to the biological interactions comprises more hydrophobic residues, such as LEU and VAL as shown in Table 5. Table 5 also indicates that the contacts of identical residues easily occur in obligate interactions. However, this is found less in non-obligate interactions as shown in Table 6 and Table 7.

**Figure 1:**
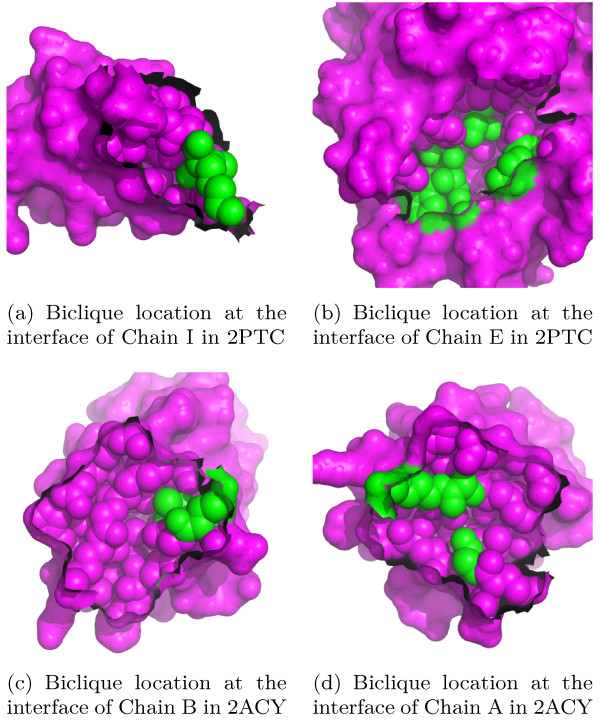
**Examples of biclique location at the protein interfaces**. All residues in this figure are shown in 'spheres' and 'surface' view with the magenta color; the biclique residues are in green; the parts without 'surface' view are the interfaces; the complexes of each row can be constructed by rotating the left figure counterclockwise and the right clockwise round the axis of the middle vertical line.

We would also like to present the conservation scores and the residues' ASA (*accessible surface area*) of unique bicliques in specific PDB entries. We take  in the crystal packing 2ACY in Table 8 and  in the transient interaction 2PTC in Table 9 as example. In these two tables, the conservation score is taken from the website of rate4site [31], while ASA is calculated by NACCESS [32]. The location of these two bicliques at the protein interfaces are displayed in Figure 1.

**Table 8:** The conservation score and ASA (Å) information of the unique biclique  = ⟨{ARG, GLY, SER},{ASN, LYS}⟩ in PDB entry 2ACY

**Pos**.	Residue	Cons. Score	ASA in chain	ASA in complex
15	GLY	9	35.33	0.0

16	LYS	6	131.14	53.37

45	GLY	9	36.32	0.2

44	GLN	6	147.86	54.17

46	THR	7	10.97	0.0

**Table 9:** The conservation score and ASA (Å) information of the unique biclique  = ⟨{GLN, GLY, SER, SER, TYP},{CYS, LYS}⟩ in chain E and I of PDB entry 2PTC.

**Pos**.	Residue	Cons. Score	ASA in chain	ASA in complex
**residues in **2PTC** chain E**				

192	GLN	1	116.42	20.18

195	SER	9	19.16	0.0

214	SER	9	10.96	2.06

215	TRP	4	51.96	5.44

216	GLY	9	29.15	1.49

**residues in **2PTC**chain I**				

14	CYS	9	55.63	0.0

15	LYS	3	201.71	0.6

It can be seen from Table 9 that the biclique residues in  have relatively small ASA in the 2PTC complex and larger ASA change upon complex formation. This can be easily understood from Figure 1: Figure 1(a) and 1(b) clearly show that the biclique residues in the biological interface 2PTC are buried. The ASA of LYS15 in chain I decreases from 201.71 Å to 0.6 Å, indicating that this biclique is closer to the interface center than to the rim of interface in Figure 1(a). However in the crystal packing 2ACY, both partites of  have residues with relatively larger ASA, more than 50 Å. So, the biclique residues in chain B in Figure 1(c) are a little away from the interface center.

As shown in Table 8 and Table 9, both bicliques contain residues with high conservation scores. However, three of the seven residues in  have a conservation score less than 5. For example, the conservation score of LYS15 in chain I of 2PTC is 3, but this residue contributes greatly to the formation of the complex 2PTC - its mutation results in a big binding free energy change (10 kcal/mol) according to ASEdb (Alanine Scanning Energetics database) [4]. This observation might give a hint that although residue conservation is one of major factors contributing to frequent bicliques, frequent unique bicliques to biological interactions can capture more specific evidence for understanding complex formation than the conservation alone, such as ASA, residue physicochemical properties, and tightly packing residue contact. Next, we present our sequence and structural analysis results on the unique biclique . As shown in Table 7,  occurs only in six non-obligate interactions in six different PDB protein complexes. These six interactions are all about trypsins/trypsinogen interacting with different types of inhibitors in different organisms. For example, 1TGS is about 'three-dimensional structure of the complex between pancreatic secretory inhibitor (kazal type) and trypsinogen', and 2BTC is about 'bovine trypsin in complex with squash seed inhibitor (cucurbita pepo trypsin inhibitor II)'. The sequence similarities of the six interacting chain pairs are as follows. Chain E of 1TAB, chain Z of 1TGS, chain E of 2BTC and chain E of 2PTC are identical chains. In comparison to this identical chain, chain A of 1EJA has only 83% sequence similarity and chain H of 1BTH possesses only about 36% sequence similarity. The sequence similarity among the other chains of these interactions is very low except two identical chains P in 1BTH and I in 2PTC.

Overall, there are no two pairs of interactions whose sequence similarity is larger than 40%. That is, there is no pair sequence redundancy in these six non-obligate interactions. We also note that although the two chain Es in 2BTC and in 2PTC are identical chains, the specific residues involved in  are not the same due to the low similarity between their partner chains. Residue SER in 2BTC is in the position 217, while it is in the position 195 in 2PTC. So, both bicliques are interesting to show. The details of these sequence similarities are provided in Table 10. The computational steps for determining the sequence similarity between two sequence pairs can be found in **Methods**.

**Table 10:** Sequence similarity (%) among corresponding chains of the protein interaction pairs in Table 7.

*PDB entries*	1EJA***:B***		1TAB***:I***		1TGS***:I***		2BTC***:I***		2PTC***:I***		1BTH***:P***	
1EJA**:A**	**A**	*B*	-		-		-		-		-	

1TAB**:E**	**83**		**E**	*I*	-		-		-		-	

1TGS**:Z**	**83**		**100**		**Z**	*I*	-		-		-	

2BTC**:E**	**83**		**100**		**100**		**E**	*I*	-		-	

2PTC**:E**	**83**		**100**		**100**		**100**		**E**	*I*	*100*	

1BTH**:H**	**37**		**36**		**36**		**36**		**36**		**H**	*P*

The *italic *half is for the sequence similarity among these inhibitors, while the **bold-face **half is for the sequence similarity among trypsins/trypsinogen; '-' means no significant sequence alignment.

**Figure 2:**
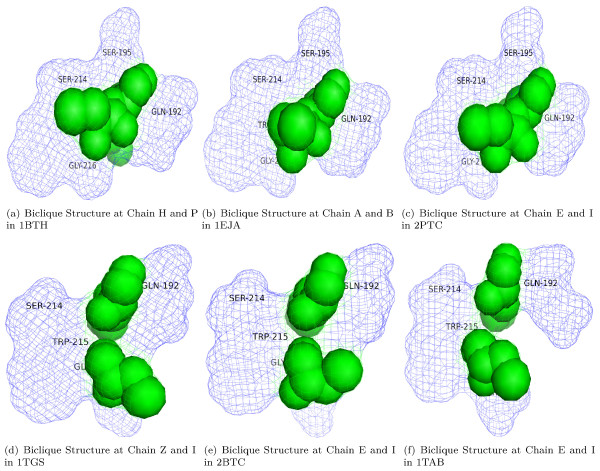
**The structure of the unique biclique  = ⟨{GLN, GLY, SER, SER, TYP}, {CYS, LYS}⟩ in six interaction chain pairs**. In these figures, residues from one chain in blue color are shown in the 'mesh' view, and residues from the other chain in green color are shown in the 'spheres' view.

The 3D structures of this DWE biclique in the six different PDB protein complexes are displayed and compared in Figure 2. The 3D shape of these structures looks highly similar to each other with a common lock-and-key topology [33]. Since this stable topology is repetitive in six non-obligate interactions, it is worthy of further investigation to see whether this group of residues in this biclique is closely related to or involved in the above mentioned protein functions.

Another interesting question is: which residues in this DWE biclique are energetically outstanding. As mentioned, Lys in the sequence position 15 of chain I in 2PTC is a wet-lab confirmed hot spot residue with an extremely high energy (10 kcal/mol) according to ASEdb [4]. This may suggest that the Lys residue is also a hot spot residue in the other 5 interacting chain pairs.

### C. Residue Composition of the DWE Bicliques for the Three Types of Protein Interfaces

**Figure 3:**
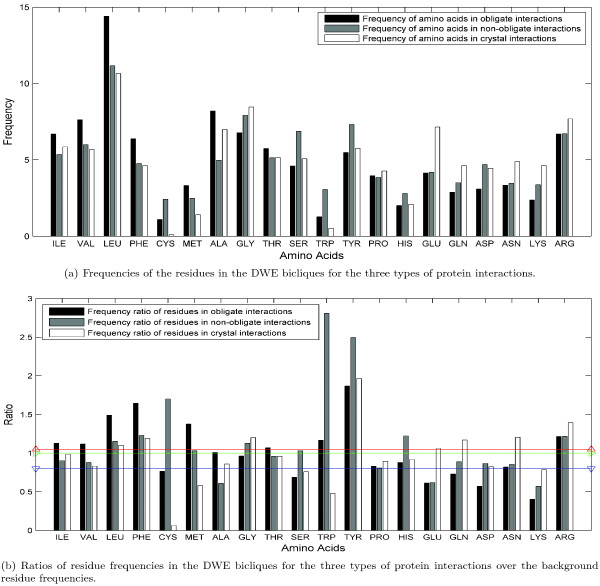
**Residue compositions in DWE bicliques are different in the three types of protein interactions: obligate, non-obligate interactions and crystal packing**. Here, the residues are ordered according to their hydrophobicity with Ile as the most hydrophobic and Arg as the least hydrophobic. The background residue frequencies are the percentage of the amino acids for the complete database from the release 55.0 of Swiss-Prot.

The residue composition of protein binding interfaces or binding sites has been intensively studied previously [13,21-24,34]. The composition of residues and residue pairs in DWE bicliques are studied by the current work in order to understand whether protein binding hot spots change their residue composition under the constraint of 'double water exclusion' hypothesis. We focus on the preference and tendency of residues to the specific types of interactions, as well as the preference and tendency of residue pairs. We would like to note that the composition of residues and their pairs in unique bicliques may be more interesting than those in DWE bicliques. But our investigation shows that there is no significant change for the composition of residues and their pairs in going from DWE bicliques to unique bicliques. This situation may be due to (i) that unique bicliques dominate DWE bicliques, and/or (ii) that common bicliques among the different types of interactions, especially those with larger support ratios, may also cover useful patterns for understanding protein binding behaviors. Therefore in the following two subsections, our analysis on the residues and their pairs in DWE bicliques is not on unique bicliques alone. We begin our analysis on the interaction-dominated residues (short for idRs) and interaction-preferred residues (short for ipRs). A residue is an idR in a type of interactions if its percent frequency in a set of DWE bicliques for this type of interactions is high; while a residue is an ipR in a type of interactions if its frequency ratio over the background is high. See **Methods **for the detailed definitions of idRs, ipRs and anti-ipRs. Figure 3(a) shows the frequency information of the twenty amino acids in our DWE bicliques for the three types of interactions, and Figure 3(b) displays the frequency ratio information of the twenty amino acids with reference to their background frequencies.

From Figure 3(a), we can see that the idRs for the obligate interactions are Leu (14.4%), Ala (8.20%), Val (7.63%), Gly (6.78%), Ile (6.68%), Arg (6.68%) and Phe (6.38%) ordered by their frequencies. Five of these residues are hydrophobic except Gly and Arg. However, Arg is broadly considered to be the richest in hot spots [1]. Gly's frequency ratio over its background percentage is 0.96, near to 1. That is, the abundance of Gly in nature makes Gly become an idR. Thus, we can make a conjecture that the binding hot spots of obligate interactions are dominated by hydrophobic residues. This point agrees to the frequency ratio trend of the ipRs of the obligate interactions as shown in Figure 3(b). The ipRs in these obligate interactions are Tyr (1.87), Phe(1.64), Leu(1.49), Met(1.38), Arg(1.22), Trp(1.17), Ile(1.13), Val(1.12) and Thr(1.07) according to the ranking of their frequency ratios. Six of these ipRs are hydrophobic except Tyr, Arg and Thr. However, Tyr is an aromatic residue which can form *π*-*π*/cation-*π *interactions to stabilize protein binding. This is also why all aromatic residues, Tyr, Phe and Trp, are ipRs of obligate interactions. Another interesting observation from Figure 3(b) is that the anti-ipRs are Cys, Gln, Ser, Glu, Asp and Lys-none of them is hydrophobic. Therefore, all these observations are consistent, and indicate that the binding hot spots of obligate interactions are hydrophobic and stable.

Different from the obligate interactions, the binding hot spots of the non-obligate interactions contain only three hydrophobic idRs (Leu, Val and Ile), in addition to three hydrophilic idRs (Gly, Tyr and Ser) and one basic idR (Arg). It seems that non-obligate interactions are generally less hydrophobic than obligate interactions. The ipRs of these non-obligate interactions have a similar composition to their idRs, including three hydrophilic residues (Trp, Phe and Leu), three hydrophobic residues (Tyr, Cys and Gly), and two basic residues (His and Arg). Three aromatic residues, especially Trp and Tyr, seem to have a higher propensity to non-obligate interactions.

The idRs of crystal packing are Leu (10.65%), Gly (8.46%), Arg (7.68%), Glu (7.16%), Ala (6.98%), Ile (5.85%) and Tyr (5.76). But the anti-ipRs of crystal packing, such as Met, Trp and Cys, become ipR residues of obligate or non-obligate interactions. As expected, biological interactions have different residue preference from crystal packing in their DWE bicliques.

#### Quantifying the Difference of Residue Composition for the Three Types of Protein Interactions

We take two ways to quantify the residue composition difference between different interaction types. One is a Euclidean distance Δf [3,13] as described by Equation 1 in **Methods **to measure the difference of residue percent composition in the three types of protein interactions; the other is a correlation coefficient *CC *[20] as described by Equation 2 in **Methods **mainly to compare different residue ratio composition. The comparison result is presented in Table 11.

**Table 11:** The difference of residue composition in the three types of protein interactions.

Interaction Types	Obligate	Non-obligate	Crystal Packing
Obligate		*0.5219*	*0.4640*

Non-obligate	2.56%		*0.0561*

Crystal Packing	2.87%	2.06%	

Background	3.70% (*0.78*)	2.922% (*0.70*)	1.52% (*0.88*)

The *italic *numbers are for correlation coefficient, and others for Euclidean distance.

It is not surprised to see that the residue composition of all the three types is highly correlated to the background residue composition in the Swiss-Prot database with *CC *> 0. 7 [20]; however, biological interactions have larger Euclidean distance from the background residue composition with Δf = 3.7% for the obligate interactions and Δf = 2.922% for the non-obligate interactions. The Euclidean distance of residue percent composition in the three types of interactions is also large. For example, this Euclidean distance between the obligate and non-obligate interactions is 2.56%, while that between the cores of protein-protein complexes and homodimers is 2.0% [13].

We can understand from Table 11 that frequency ratio of residue composition in the three types of protein interactions has very low correlation coefficient, especially between non-obligate interactions and crystal packing with *CC *= 0.0561. The exceptionally larger correlation coefficient but larger Euclidean distance (Δf = 2.87%) between crystal packing and obligate interactions is partly, if not mainly, due to that most of crystal packing in the analyzed dataset are based on identical chains while most of obligate interactions are homodimers.

#### Comparison on Residue Composition Between the O-ring and DWE Hypothesis

Our analysis result on the residue composition of our DWE bicliques is in agreement with the influential study by Bogan and Thorn [1] who investigated the binding hot spots of protein interfaces under the O-ring hypothesis. Bogan and Thorn [1] found that hot spots are abundant with Trp, Tyr and Arg. We also found that these three residues are actually ipRs for both obligate and non-obligate interactions. Similar to Bogan and Thorn's method, Janin and her colleagues had a study for identifying the core and rim from a protein interface. They found that aromatic residues have high propensity values in the core of protein-protein recognition sites [3]. For homodimeric proteins, their another work [13] pointed out that aliphatic and aromatic residues are very rich in the binding hot spots. All these results are consistent with ours.

Therefore, we can see that when protein binding hot spots are refined from the O-ring theory to the double water exclusion hypothesis, the composition properties are inherited and some properties are more enlightened and sharpened.

### D. Residue Pairing Preference in DWE Bicliques for the Three Types of Protein Interfaces

A DWE biclique can contain many residue pairs. We are interested in those residue pairs that dominate, with high frequency, the binding hot spots of a type of protein interactions. We term this kind of residue pairs as interaction-dominated residue pairs (or idRPs for short). Meanwhile, we also examine interaction-preferred residue pairs (ipRPs). (See exact definitions for idRPs and ipRPs at the **Methods **section.)

**Figure 4:**
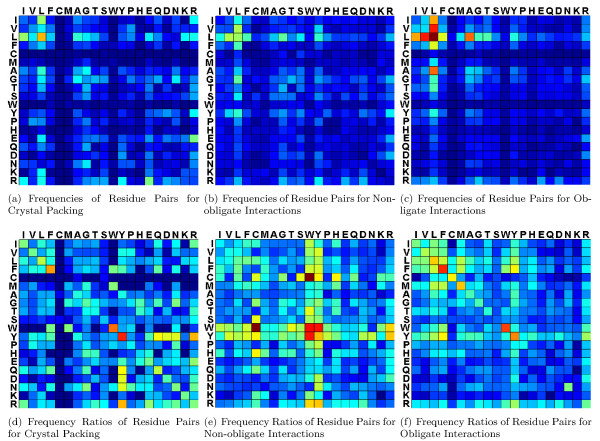
**Residue pair composition in DWE bicliques for crystal packing, non-obligate interactions and obligate interactions**. (a), (b) and (c) show the frequency matrixes of residue pairs in these three types of interactions; (d), (e) and (f) are the matrixes of natural logarithm of frequency ratios for residue pairs in these three types of interactions. These figures are symmetric matrixes of residue pairs where rows and columns represent different amino acids, and the residues are ordered according to their hydrophobicity with I as the most hydrophobic and R as the least hydrophobic. In these figures, the colors from blue to red mean the values from smallest to largest, and the similar colors mean the similar value in the second row.

The composition information of all possible 210 residue pairs in our DWE bicliques is displayed in Figure 4. It can be seen that the obligate interactions are dominated by the contacts of hydrophobic residues. Taking the idRP group of I-V-L-F as example, the total frequencies of ten idRP contacts within this group are 18.22%, 10.74% and 11.74% for the obligate interactions, non-obligate interactions and crystal packing, respectively. The contact frequency of this most hydrophobic group in the obligate interactions is much higher than those in the other two types of interactions.

The ipRPs sharpen the difference of residue pairs in the three types of interactions. In the obligate interactions, most ipRPs are from four groups: (i) the contacts of identical residues, especially the residues with hydrophobicity not less than Tyr (Y)-this observation agrees with the discussion in [24] where identical residues more likely contact themselves in obligate interactions; (ii) the interacting pairs between aliphatic residues, Ile (I), Val (V) and Leu (L)-all these residues are most hydrophobic; (iii) the contacts between aromatic residues, Tyr (Y), Trp (W) and Phe (F); and (iv) the contact pairs between aromatic residues and Arg(A)/aliphatic residues. Aromatic residues are much involved in ipRPs due to that they easily form *π*-*π*/cation-*π *contacts which are vital to the stability of biological interactions. Besides the above ipRPs, another three ipRPs of obligate interactions are residue pairs Met(M)-Leu(L), Ala(A)-Leu and Met-Phe(F).

All these ipRPs shape an interesting distribution as outlined in Figure 4(f). Most of the ipRPs are located at the top-left corner of Figure 4(f), an area on the top of and at the left of Tyr (Y) included. We call this area ipRP area. There are fewer ipRPs outside this area, while those ipRP exceptions outside the ipRP area are the identical contacts of Asn (N) and Arg (R), and the interactions between Arg (R) and aromatic residues. That is, when taking the aromatic residue Tyr (Y) as the dividing line for the columns and also for the rows in Figure 4(f), ipRPs are depleted in the top-right corner (and also the bottom-left corner due to the symmetry of Figure 4(f)). The bottom-right corner also has rare ipRPs where the least hydrophilic residues are solely involved in the contacts. Such an ipRP distribution suggests that it is the very hydrophobic contacts that much prefer to the obligate interactions.

Similarly, in the non-obligate interactions, there are also very fewer ipRPs in the bottom-right corner of Figure 4(e) except the contact between Arg (R) and Asp (D). In the non-obligate interactions, the ipRPs are mainly from the contacts involving Trp (W), Tyr (Y), Phe (F), Cys (C), His (H) and Leu (L), specially the contacts involving W and Y. Of these contact residues, three are aromatic residues (Y, W and F), and two are nonpolar (hydrophilic C and hydrophobic L). Cys-involved ipRPs are expected due to that Cys contains a sulfate atom and can form disulfate bridges to stabilize the protein interactions. The reason why H is also involved in the ipRPs may be that H is sometimes categorized into aromatic residues [35] and likely possesses some properties of aromatic residues in certain environments. In conclusion, residue pairs involved by hydrophobic and aromatic residues are abundant in the two types of biological interactions, indicating the importance of these ipRPs in specific binding behaviors.

In contrast to biological interactions, the ipRP distribution for the crystal packing contacts is completely opposite. Crystal packing contacts have more ipRPs of hydrophilic contacts (at the bottom-right corner of Figure 4(d)) and fewer ipRPs at the top-left corner. The top-right corner (and also the bottom-left corner due to the symmetry) of Figure 4(d) has more ipRPs than the top-left corner does.

## Conclusions

With the integration of the influential O-ring theory and the insightful 'coupling proposition', DWE (double water exclusion) is a more comprehensive hypothesis for modeling protein binding hot spots. In this work, we constructed DWE bipartites from interacting protein chains under the constraints of both residue contacts and residue accessibility. Biclique patterns were then detected for each type of protein interactions. Our comparative analysis on DWE bicliques suggested that there do exist unique bicliques in the three types of interactions. Compared to crystal packing, those unique bicliques only occurring to biological interactions made it much clear that the biological binding behaviors have strong specificity. The unique bicliques in the obligate and non-obligate interactions also confirmed the different binding behaviors in these two types of biological interactions. Therefore, the idea of DWE bicliques provides a new way to the study on protein interfaces.

The composition of residues and the composition of residue pairs, in particular ipRs and ipRPs, did reveal the deep characteristics of these types of interactions. The protomers of obligate interactions fold and bind at the same time. Obligate interfaces need hydrophobic residues to form their interior cores, similar to the cores of protein tertiary structures in the same folding-binding process. Therefore in the obligate interactions, hydrophobic residues were greatly involved in ipRs and ipRPs, while none of the anti-ipRs of the obligate interactions was hydrophobic. Also in this process of protein folding and binding in a solvent environment, hydrophilic and polar residues prefer protein solvent surface than hydrophobic core, and the contacts involved by hydrophilic residues of obligate interactions were thus depleted in ipRPs.

On the other hand, two protomers in non-obligate interactions fold separately. They then come together to bind upon a specific molecular stimulus, and may dissociate after that. In a unbound form of non-obligate protomers, their interface surfaces have to contact with the solvent, and less hydrophobic residues are necessary [23] to keep the stability of unbound non-obligate protomers. So, the hydrophobic ipRPs in non-obligate interactions are much less than in obligate interactions. To compensate for the decrease of hydrophobic ipRPs in non-obligate interactions, aromatic residues are rich in non-obligate interfaces. Aromatic residues, such as Trp, Tyr and His sometimes, can contribute protein binding through the hydrophobic effect. Meanwhile, aromatic residues do not result in a large entropic penalty because they have few rotatable bonds [1]. This is why aromatic ipRs and their ipRPs were observed to be abundant in biological interactions, especially in non-obligate interfaces.

In contrast, hydrophilic ipRs and ipRPs were affluent in crystal packing to stabilize monomers in crystal packing without interactions. The anti-ipRs of crystal packing, such as Met, Trp and Cys, were just the ipRs of the biological interactions, indicating they can form significant atom contacts to greatly increase the probability of biological interactions, such as *π *involving contacts and disulphide bridges.

In summary, these ipRs and ipRPs in DWE bicliques are excellent indicators for the specificity analysis of biological binding behaviors. They can be used to identify biological interactions from crystal packing and classify different types of biological interactions, such as obligate and non-obligate interactions [30]. The identification of obligate or non-obligate interactions can help docking algorithm to remove the noise of produced crystal packing.

## Methods

### A. Compiling a Nonredundant Dataset

The data used in this paper contains three types of protein interactions: obligate interactions, non-obligate interactions and crystal packing contacts. All of them are obtained from previously published literature works. (i) The obligate interactions are from the obligate interactions used in [26,27], as well as the homodimers used in [22,25]; (ii) the non-obligate interactions comprise the non-obligate interactions used by [26,27], and the protein complexes used in [22]; and (iii) the crystal packing contacts are those from [22,26] and the monomers used in [25].

To get rid of the redundancy within each type of protein interactions, we remove those redundant interaction pairs with high similarity. Let  and , and  and , *i *≠ *j*, be two pairs of protein interaction chain pairs in one type of interactions, these two pairs are redundant if *score * and *score*, or *score * and *score*, where the *score *function is a sequence similarity score of two protein sequences and it can be produced by the BLAST software (downloadable from NCBI http://www.ncbi.nlm.nih.gov/BLAST/download.shtml) without filtering of low compositional complexity, and *s *= 90% here. This redundancy removing process resulted in a non-redundant data set comprising 291 crystal packing contacts, 289 non-obligate interactions and 287 obligate interactions. The distribution of these interactions under different *s *value ranges is shown in Table 12. At one hand, it is clear in Table 12 that most of them have a low similarity of *s *= 40% or below. On the other hand, the detected bicliques from chain pairs with high similarity are actually different. For example, in Table 7, although chain E of 2PTC and chain E of 1TAB are the identical, the occurring bicliques are involved with residues of different positions in the interaction partner chains.

**Table 12:** The chain-pair distribution in our nonredundant dataset according to the similarity.

	Similarity region						
	
	(80,90]	(70,80]	(60,70]	(50,60]	(40,50]	[0,40]	Total
Obligate	0	1	1	3	11	271	287

Non-obligate	3	4	6	5	15	256	289

Crystal Packing	0	3	1	4	9	274	291

### B. Constructing DWE Bipartites for Protein Interactions

Given two interacting polypeptide chains *C*_1 _and *C*_2_, according to the DWE hypothesis, we define its DWE bipartite as a bipartite graph *G *= ⟨*V*_1_, *V*_2_, *E*⟩, where (i) the vertices in *V*_1 _and in *V*_2 _represent the amino acids from *C*_1 _and *C*_2 _respectively; (ii) the relative accessibility of all residues in *V*_*i*_, i = 1, 2, is less than a certain threshold *t*_*ra*_; and (iii) *E *represents all residue contacts between *V*_1 _and *V*_2_, and every residue in *V*_*i *_must contact at least one residue in *V*_*j*_, i, j = 1,2 and i ≠ j.

We take two steps to construct DWE bipartites: (i) constructing bipartite graphs from protein interactions; (ii) filtering out those residues in the bipartite graphs by using the constraint of residue accessible surface area.

#### Constructing Bipartite Graphs

Each pair of chains can be transformed into a bipartite graph according to the contact requirement of the DWE bipartites above. In this work, two amino acids from *V*_1 _and *V*_2 _are considered as contact if the minimum of the distances of atoms from these two amino acids is less than the sum of van der Waals radii of the corresponding atoms plus a certain threshold. To ascertain that there is no water between interacting residue pairs, this threshold, denoted as *d*_*water*_, is set to van der Waals diameter of water molecules (2.75 Å).

In other words, residue *r*_*ik *_of *C*_*i *_and residue *r*_*jl *_of *C*_*j*_, *i*, *j *= 1, 2 and *i *≠ *j*, contact if and only if the minimal distance among those distances between the atoms of *r*_*ik *_and the atoms of *r*_*jl *_is less than *d*_*water*_. Here, all heavy atoms in backbone and sidechains of amino acids are used. The distance between a pair of atoms *a*_*i' *_from *r*_*ik *_and *a*_*j' *_from *r*_*jl *_is calculated by: *d *= *d*(*a*_*i'*_, *a*_*j'*_)-*r*(*a*_*i'*_)-*r*(*a*_*j'*_) where *d*(*a*_*i'*_, *a*_*j'*_) is the spatial distance of *a*_*i' *_and *a*_*j'*_, and *r*(*a*_*k'*_) is van der Waals radius of *a*_*k'*_, *k' *= *i' *or *j'*. Suppose we are given *m*_*p *_number of protein interactions, the bipartite graph database can be denoted by  where *G*^*i *^represent the protein interactions.

#### Accessibility Filtering

The constructed bipartite graphs *P *are further processed by using the constraint of water accessible surface area of residues. We take NACCESS [32] to produce the relative accessible surface area for each residue in a protein interaction. The remaining ones are only those residues whose relative accessible surface area is less than a certain threshold *t*_*ra*_. In this work, *t*_*ra *_is set as 36% as recommended by [9]. The resulting bipartite graphs are called DWE bipartites of protein interactions, denoted by

### C. Mining Maximal DWE Bicliques From DWE Bipartites

A biclique is a special bipartite graph where each residue in one partite contacts with every residue in the other partite. A DWE biclique is a biclique from a DWE bipartite. Maximal DWE bicliques  = ⟨*V*_1_, *V*_2_, *E*⟩ are DWE bicliques where there is no other DWE biclique  containing . In this work, maximal DWE bicliques  are abbreviated to  = ⟨*V*_1_, *V*_2_⟩ without *E *due to the constraint of all-versus-all interactions between the residues in *V*_1 _and *V*_2_. In protein interactions, a maximal DWE biclique  represents densely interacted residue pairs in a compact region.

Given a DWE bipartite , we take the LCM-MBC algorithm [36] to mine maximal DWE bicliques. The LCM-MBC algorithm needs two parameters: *p *and *q*, *p *≤ *q*. Suppose that *V*_*i *_is with less vertices than *V*_*j *_in a maximal DWE biclique , *i*, *j *= 1 or 2 and *i *≠ *j*, *p *is the minimum size of *V*_*i *_and *q *is the minimum size of *V*_*j*_. That is, the LCM-MBC algorithm filters out those  in which the minimum size of *V*_*i *_is less than *p *or the minimum size of *V*_*j *_is less than *q*. In this work, *p *is set to 2 and *q *to 3. Assume that the LCM-MBC algorithm detect *n *maximal bicliques from *H*, denoted as *M *= { = ⟨*V*_1_, *V*_2_⟩ |*j *= 1, 2, ..., *n*} where items  are maximal bicliques with amino acids as their vertices. Not every  in *M *is useful for our analysis due to that some bicliques are infrequent and random.

Therefore, for each DWE biclique , we enumerate *H *to get its occurrence in protein interactions. If the occurrence is not less than a threshold *sup*,  is considered to be interesting. Here, a biclique occurs in an interaction if all the residues in this biclique are in the DWE bipartite of this interaction and these residues also maintain the same biclique structure of full contacts. However, the space of possible bicliques is too large. Take bicliques of one partite with 2 residues and of the other partite with 3 residues for example, there are 323,400(( + 20) × ( + 20^2^)) possible bicliques and 20^5 ^biclique instances if each residue is considered to be independent. However, there are only 868 interactions including non-biological interactions. Thus, if each residue is with equal probability, the maximum of the expected support levels for these bicliques is 0.027 (10*868/20^5^), much less than 1. That is, those bicliques whose support is equal to 1 also have a higher support than what they are expected. They have a lower support likely due to that there are limited sample interaction pairs and larger biclique space. However, bicliques occurring once can not show the specificity of binding behaviors in different types of protein interactions. Therefore, *sup *is set to 2 in this work, and the frequent maximal bicliques are referred to as DWE bicliques.

### D. The Definitions Related to Our Composition Analysis on Residues and Residue Pairs

We calculate the frequencies of residues and residue pairs in DWE bicliques for each type of protein interactions. We define interaction-dominated patterns, idR and idPR, as follows. Given an amino acid in one type of interactions, if it is at the top of residue rank in the frequency descending order, this amino acid maybe contributes more to this type of protein interactions and is defined as an interaction-dominated residue (idR). In this work, idRs are defined as the top seven residues (a little more than one third of all the twenty standard residues). If a pair of contact residues has top frequencies in one type of protein interfaces, this pair is termed as an interaction-dominated amino acid pair (idRP).

Interaction-dominated patterns alone might not clearly exhibit the preferred ways for different types of interactions, as the percentages of the twenty standard residues in nature are not equal. Thus, we define interaction-preferred patterns to help the dissection analysis on protein interfaces. Assume that the percentage of amino acids for the complete database from release 55.0 of the Swiss-Prot database http://cn.expasy.org/sprot/relnotes is our background residue composition, then

(i) we define a residue as an **ipR **in one type of interactions, if the frequency of the residue is higher than its frequency in the background residue composition. As this animo acid prefers to this type of protein interactions, we name it an interaction-preferred residue (ipR). In this work, the extent of such preference is measured by the ratio of the residue frequencies in each type of protein interfaces over the corresponding residue percentage in the background composition. This ratio can indicate how much the residues prefer a certain type of protein interfaces. Thus, in a given type of protein interactions, if the ratio of a residue is greater than a ratio threshold, this residue is an ipR. To reduce the influence of random errors, this ratio threshold here is set to 1.05 rather than 1. In contrast, the residues whose ratios are less than another ratio threshold are called anti-ipR residues.

In this work, the second ratio threshold is set to 0.8. Please note that Ofran and Rost [20] also took the percentage of amino acids from the complete database of the Swiss-Prot database as a background residue composition to measure the preference of residues.

(ii) we define a residue pair as an interaction-preferred residue pair (**ipRP**) if they have much higher frequencies than random cases. In this work, the frequencies of residue pairs (*r*_*i*_, *r*_*j*_) in the random case are calculated as  where  and  are the background percentage of residues *r*_*i *_and *r*_*j*_. Here, there is an assumption in the random case: each amino acid occurs in protein interfaces independently. Similar to the definition of ipRs, frequency ratios of residue pairs are calculated by the frequencies of residue pairs in each type of interfaces over those in the random case. These ratios can suggest the specificity of ipRPs in the different types of interactions. However, there are too many ipRPs. In this work, we focus on the residue pairs with top highest frequency ratios, and ipRPs only refer to as those residue pairs whose natural logarithm of *r*+1 is larger than 1.05 where *r *is their frequency ratio.

We would like to point out that only patterns in biological interactions are interesting, and patterns in crystal packing are used as reference for comparison.

### E. Measures for Comparison Analysis on the Residue Composition Differences

In this work, we employ Euclidean distance Δf as defined in [3,13] to measure the difference of residue percent compositions in different types of protein interactions:(1)

where *f *and *f' *are percent composition of the twenty standard amino acids. Meanwhile, correlation coefficient [20], *CC*, is also used to compare residue ratio compositions:(2)

where *r*^*x *^and *r*^*y *^are frequent ratio vector for the twenty standard amino acids, and  is the mean of the corresponding *r*_*i*_s.

## Authors' contributions

QL implemented the mining process and performed the statistical analysis. JL participated in the design and analysis and supervised the project. QL and JL drafted the manuscript together. Both authors read and approved the manuscript.
